# Virtual Vibrational Spectrometry of Stable Radicals—Necklaced Graphene Molecules

**DOI:** 10.3390/nano12040597

**Published:** 2022-02-10

**Authors:** Elena F. Sheka

**Affiliations:** Institute of Physical Research and Technologies, Peoples’ Friendship University of Russia (RUDN University), 117198 Moscow, Russia; sheka@icp.ac.ru

**Keywords:** virtual vibrational spectrometry, virtual spectrometer, semiempirical Hartree–Fock approximation, digital twins, necklaced graphene molecules, *sp*^2^ amorphous carbons

## Abstract

The article presents results of an extended virtual experiment on graphene molecules performed using the virtual vibrational spectrometer HF Spectrodyn that exploits semiempirical Hartree–Fock approximation. The molecules are composed of flat graphene domains surrounded with heteroatom necklaces. Not existing individually, these molecules are met in practice as basic structure units of complex multilevel structure of all *sp*^2^ amorphous carbons. This circumstance deprives the solids’ *in vitro* spectroscopy of revealing the individual character of basic structural elements, and *in silico* spectrometry fills this shortcoming. The obtained virtual vibrational spectra allow for drawing first conclusions about the specific features of the vibrational dynamics of the necklaced graphene molecules, caused by spatial structure and packing of their graphene domains as well as by chemical composition of the relevant necklaces. As shown, IR absorption spectra of the molecules are strongly necklace dependent, once becoming a distinct spectral signature of the amorphous body origin. Otherwise, Raman spectra are a spectral mark of the graphene domain’s size and packing, thus disclosing the mystery of their universal D-G-band standard related to graphene-containing materials of various origins.

## 1. Introduction

The concept of virtual spectrometry has arisen recently, but it has turned out to be attractive and productive for placing things in order in the field of computational spectroscopy. A virtual spectrometer, as any device, is usually designed to carry out a large number of measurements. Certainly, these measurements’ accuracy is limited by the device’s technical characteristics. Whatever these characteristics are, there will always be reasons for their inconsistency, with some specific requirement of a real experiment. Underlying computational software determines the technical characteristics of a virtual spectrometer. In the case of vibrational spectrometry, the programs must allow for solving the dynamical problem of an object using the methods of quantum theory. Thus, in the first realized virtual multi-frequency spectrometer (VMS) [1], the calculations were carried out using available CCSD(T) and DFT codes, supplemented with VPT2 approximation concerning dynamical problems. In the second case of the spectrometer HF Spectrodyn [2], the calculations were performed in the Hartree–Fock (HF) approximation using its either restricted (RHF) or unrestricted (UHF) versions. The result of the five-year use of the VMS [3] allowed for the clarification of the requirements for basic programs that would provide a close agreement of the virtual and experimental vibrational and electronic spectra. This was solved for small molecules. In the second case, the main attention was directed to the calculation of the vibrational spectra of large-size molecules, including molecular radicals, carried out using semiempirical UHF approximation. In contrast with the former case, the basic computational program does not foresee correction to the force matrix elements, due to which an accurate fitting of virtual and experimental spectra could not be provided and thus was not a target. The calculations were concentrated on revealing general trends under the condition of a reliable similarity of general structural images of virtual and experimental spectra.

The first goal of the virtual vibrational spectrometry of complex molecular objects [2,4,5] was mainly a new possibility to enrich information obtained from the analysis of experimental spectra. However, the proposed concept turns out to be much broader than the applied analytical tasks. Virtual spectrometry, similar to any other one, is a self-sufficient scientific field with its own skill and fundamentally new objects presenting molecular models. Obviously, these objects are not new and have been widely used in chemistry since the first structure image was suggested. What is new is their role in the modern chemistry world. Until recently, molecular models, such as the famous formula C_6_H_6_, had not lived their own lives, but have been considered as an integral accessory when describing real molecules once identified with the latter. Moreover, even the presence of a set of possible structures, such as Kekule ones, did not violate the general perception of the benzene molecule in the form of a hexagonal structure formed by C-H units, taken as a real picture. However, evidently, C_6_H_6_ hexagon is a model of a real substance called benzene.

Virtual spectrometry changes this convenient role of the molecular models, shifting the emphasis from them as representatives of the substance under study only while placing them in the position of independent objects. This circumstance logically places virtual spectrometry on par with the processes of overall digitalization, being the general trend of the current modern development (see reviews [6,7,8]). Developed independently, this accelerating trend is based on a broad concept of *digital twins* (DTs), which are defined as virtual representations of physical assets enabled through data and simulators for real-time prediction, optimization, monitoring, controlling, and improved decision making [7]. Evidently, molecular models of virtual spectrometry worthily acquire the DT status and together with spectrometers present one of standard forms of the digitalization concerning molecular chemistry R&D in the following concise scheme:

Digital twins→Virtual device→IT product

Here, DTs and virtual device present molecular models and codes of a selected software, respectively. The IT product covers a large set of computational results related to the DTs under different actions in light of the soft explored. Altogether, the scheme presents the algorithm of the digitalization of a particular branch of molecular science.

DT-based virtual vibrational spectrometry makes its first steps. Comparative analysis is its strong point, as was demonstrated on the example of polycyclic benzenoid-fused hydrocarbons [2], fullerene C_60_ and its isomers [3] as well as C_60_ dimers [4]. Conversely, the comparison of obtained virtual spectra with experimental data is the method’s vulnerability. The vast experience of all previous computational spectroscopy shows that even when the complete correspondence of the molecular model to the real object seems doubtless, in the modern molecular theory, there are no methods allowing one to obtain a complete agreement between experimental and calculated data. Any agreement on this field results from fitting the calculation parameters. As a result, the vibrational virtual spectrometry motto is *not a spectral analysis of individual modes and bands, but trends.* This approach relieves the researcher from the need to take into account all the details of the experiment and makes it possible to self-consistently establish the relationship between the initial and/or modified structure of the modeled molecule and the spectral vibrational response within the framework of the theoretical concept used. For its part, trends are always more stable in experimental spectra, such that by agreement or disagreement between the calculated and experimental trends, one can judge the correctness of the choice of the theoretical approximation. From this point of view, UHF, DFT, CI, and so on virtual spectra are analogs of experimental ones recorded on different hard spectrometers, the analysis of which should take into account the individual characteristics of the device.

The current work presents one more justification of the discussed above related to a particular class of graphene molecules. The virtual device HF Specrodyn, implementing the software CLUSTER-Z1 [9], provides the calculation of harmonic one-quantum spectra of IR absorption and Raman scattering. The calculations are based on the standard rigid-rotor harmonic-oscillator model in the framework of semiempirical HF quantum chemical approaches. A detailed description of the code can be found in [10]. All the calculations, discussed in the current paper, were performed in the UHF approximation using AM1 version of the code. Orienting on large molecules with a large number of vibrational modes, the spectrometer is aimed at not only scrupulous analysis of individual vibrational modes and their assignment to characteristic *general frequencies* (GFs), widely applied in standard molecular vibrational spectroscopy [11,12], but also at revealing general trends distinctive for the ensemble of modes of the molecules of a particular class. Throughout the paper, the virtual spectra are presented by stick-bars convoluted with a Gaussian bandwidth of 10 cm^−1^. Intensities are reported in arbitrary units, normalized per maximum value in each case. When the number of vibrational modes comprising the spectrum under consideration is too large, the excessive fine structure, statistically suppressed in practice, is covered by trend lines averaged over the 50 next steps of 0.003 ÷ 0.010 cm^−1^ each. The procedure was applied to all Raman scattering plots, but only partially to IR spectra.

This paper is composed in the following way. Digital twins of a selective set of graphene molecules are presented in Section 2. Vibrational spectra of bare graphene domains of the molecules are discussed in Section 3. The virtual spectrometry of graphene hydrides and oxides is considered in Section 4 and Section 5, respectively. Section 6 presents a discussion of the results obtained with respect to *sp*^2^ amorphous carbons. A general discussion is finished in Section 7 with a summary and conclusions.

## 2. Digital Twins of Necklaced Graphene Molecules

Bare nanosized honeycomb sheets (graphene domains below), surrounded with heteroatom necklaces of different chemical composition, were selected for the current study. These molecules are radicals [13], among which bare graphene domains are the strongest. The latter do not exist either in nature or in a laboratory, which does not prevent them from being favorite objects in virtual graphenics [14]. In contrast, necklaced graphene domains are stable radicals and are largely known as basic structure units (BSUs) of *sp*^2^ amorphous carbons, largely distributed in nature as well as in mass production in laboratories and industry [15]. The matter particularly concerns such a widely popular high-tech material as reduced graphene oxide (rGO). Existing in amorphous solid media, the BSUs vary in shape and chemical composition due to which experimental vibrational spectra of the relevant solids give only averaged presentation of the dynamics of the latter. However, recent studies of the structure and chemical composition of a set of *sp*^2^ amorphous carbons, supplemented with their neutron and photon scattering spectra as well as IR absorption and XPS study [16,17,18,19], allowed for the suggestion of reliable DTs of the relevant BSUs and has posed questions about their vibrational signatures.

**Figure 1:**
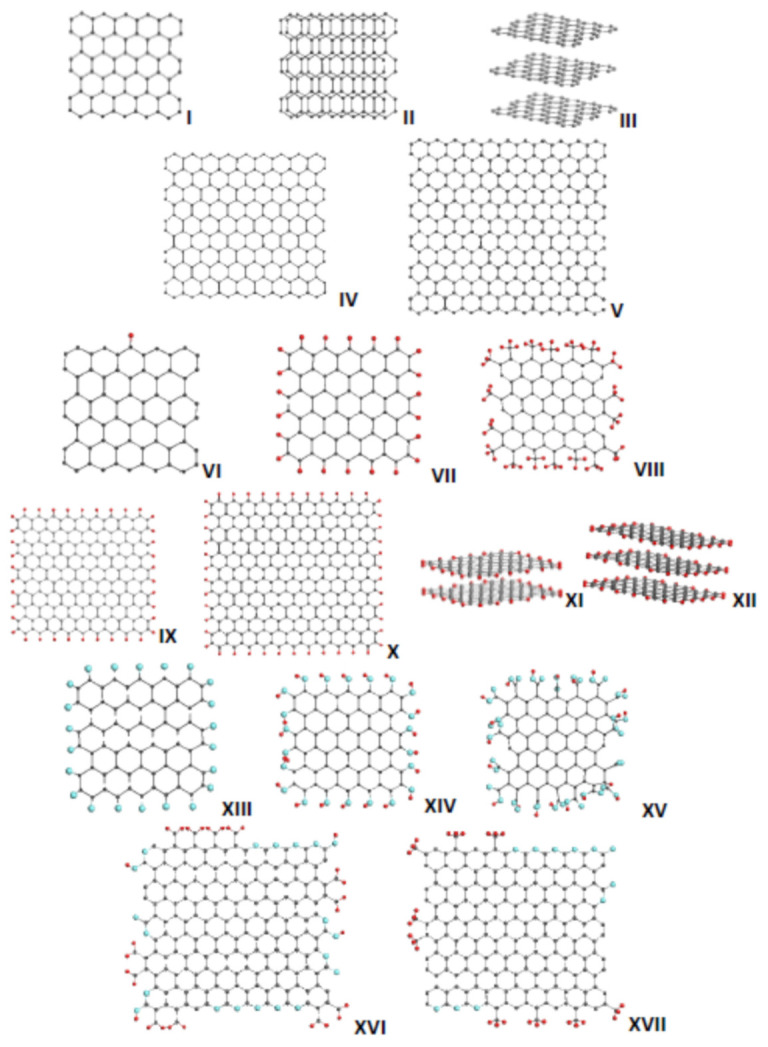
Equilibrium structures of the digital twins of the necklaced graphene molecules under study: (**I**)—one-layer, (**II**)—two-layer, and (**III**)—three-layer bare (5, 5) graphene domains; (**IV**)—(9, 9) and (**V**)—(11, 11) graphene domains; (**VI**–**VIII**) necklaced graphene hydrides based on (5, 5) domain; (**IX**,**X**)—hydrides based on (9, 9) and (11, 11) domains, respectively; (**XII**)—two-layer hydride (**VII**); (**XIII**–**XV**)—necklaced graphene oxides based on (5, 5) domain; (**XVI**,**XVII**)—necklaced graphene oxyhydrides based on (9, 9) domain and presenting BSUs of the TE-rGO (benzyl radical character of the species necklace occurs more evident than benzyl radical alcohol. as suggested earlier [17]) and Ak-rGO synthetic reduced graphene oxides, respectively (see text for details). Dark gray, red, and blue balls mark carbon, hydrogen, and oxygen atoms. UHF AM1 calculations.

Figure 1 presents DTs of a selected set of necklaced graphene molecules to be the objects of the current HF Spectrodyn experiment. The first row in the figure is occupied with DTs **I**, **II**, and **III** related to one-, two-, and three-layer stacks of the (5, 5) graphene domain. The second row presents TDs **IV** and **V** that are (9, 9) and (11, 11) graphene domains, respectively. The two next rows are given to necklaced graphene hydrides, among which TDs **VI-VIII** present hydrogenated (5, 5) domains, while DTs **IX** and **X** are analogues of TD **VII** but based on (9, 9) and (11, 11) domains. DT **XI** and **XII** present two- and three-layer stacks of TD **VII**. The next row involves three necklaced graphene oxide DTs **XIII**-**XV**. The atomic necklaces of DTs **VI**, **VII**, **VIII**, **XIII**, **XIV**, and **XV** are configured in the course of sequential step-by-step polyderivatization of the (5, 5) graphene domain described in detail elsewhere [20,21], while those of TDs **IX** and **X** are configured artificially aiming at the maximum atomic content. Additionally, DTs **XVI** and **XVII** correspond to necklaced graphene oxyhydrides based on the (9, 9) graphene domain, the size and atomic content of which were empirically determined [16,17,18,19] for two rGOs, namely TE-rGO (**XVI**) [22] and Ak-rGO (**XVII**) [23]. The choice of the (n, n) graphene domains, characterized by n benzenoid units along both armchair and zigzag edges of the honeycomb structure, was caused by two reasons. First, fully hydrogenated necklaced hydrides, similarly to DTs **VII**, at least partially, are real products obtained during the *peri*-condensation of lower acenes (see data from anthracene to pentacene [24,25]). Conversely, due to their rectangular structure, the relevant graphene domains have been favorite models (for the author of this article as well) of graphene sheets and ribbons (see [26,27,28] and references therein). Important as well is that the (5, 5) and (9, 9) domains are commensurate with BSUs of the studied *sp^2^* amorphous carbons [16,17,18,19] mentioned above.

Criterion parameters identifying the radical state of the DTs are listed in Table 1. The attribution of the obtained vibrational spectra bands is discussed in the GFs terms [11,12], a selected set of which is related to the studied compounds as listed in Table 2. Due to usual upshifting of vibrational frequencies provided with HF calculations [29], a necessary interrelation of the experimental and HF Spectrodyn frequencies data UHF Expect, attributed to the same GFs, is suggested in column 2 of the table. Here and therein the paper, all digital wavenumber notations, if not mentioned, are related to the HF Spectrodyn data.

## 3. Virtual Vibrational Spectra of Bare Graphene Domains

**Figure 2:**
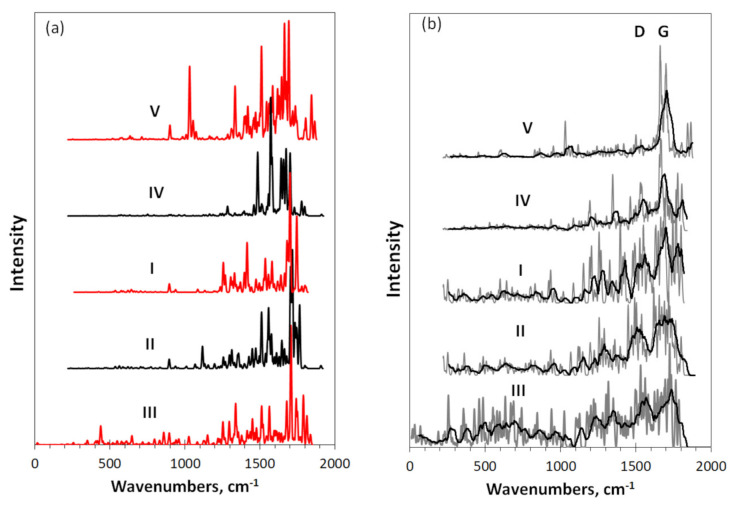
Virtual IR absorption (**a**) and Raman scattering (**b**) spectra of DTs related to bare graphene domains **I** –**V**. Digits mark DTs in accordance with Figure 1 and Table 1. UHF AM1 calculations. Two-color plotting is applied to clearly visualize intersected spectral regions.

Virtual IR and Raman spectra of DTs **I**–**V** are presented in Figure 2. The figure exhibits transformation of the vibrational spectra of the (5, 5) graphene domain **I**, which is caused by either growing its size (spectra **IV** and **V**) or stacking (spectra **II** and **III**). As follows from Table 2 and as is seen in the figure, *sp*^2^ C=C stretchings at the high-frequency edge, complemented with the ring breathings as well δ C-C-C trigonal deformations at the low-edge, are mainly responsible for the spectra shape generating a characteristic multiband structure in the region of 1200–1800 cm^−1^. This GF attribution is generally preserved, when either growing the domain size or stacking them, which is the first main trend characteristic for the species. There is a strong temptation to check the feature experimentally. However, this is not the case since the considered DTs are not related to real objects due to extremely high radicalization. The overfilled massive of the available experimental “graphene” data are not related to the bodies, but to “technical graphene” [36], which is one of either numerous rGO objects such as “graphene” quantum dots and other *sp*^2^ amorphics, nanosized units of which are heteroatom-necklaced graphene molecules described above. As we shall see later, both IR and Raman spectra of the latter depend on the necklaces’ chemical composition. Nevertheless, there are some properties of their optical spectra, common to practically all the studied graphenous materials that may shed light on a possible spectral features of bare graphene domains.

As for IR spectra in Figure 2a, the graphene domain spectrum can be traced only in the case when the heteroatom effect is small. Apparently, this happens in the case of the epitaxial grow of monolayer nanosized graphene on the SiC substrate [37] as well as occasionally occurring in the case of a large set of ceria/graphene nanocomposites [38] and graphene quantum dots [39]. As occurred, this remaining bare graphene absorption is multiband but weak, which is connected with homopolar character of the *sp^2^*C=C bonds responsible for the optical signal. As shown [40], because of nil static moment of the bonds, the case is unfavorable for a significant value of the corresponding IR intensity as well (see the discussion of DRIFT spectra of *sp*^2^ amorphous carbons [17]). This explains why the presence of any heteroatom in the graphene domain necklace drastically changes the absorptivity due to heteropolar composition of the active covalent bonds. Seemingly, the objects, selected from the sets referred above [38,39] by the lowest absorptivity, can be attributed to those disturbed by the presence of heteroatoms to the least extent.

The situation with Raman spectra is both simpler and more complex. It is simpler since the intense Raman scattering dominate from the majority of the object carbon atoms, whichever heteroatom content is in reality. This feature preliminary provides an extended standardization of the Raman spectra of any *sp*^2^ amorphous material. It is the case and the characteristic doublet of D and G bands in the 1200–1700 cm^−1^ region is the standard marker of the graphene origin of any *sp*^2^ amorphous carbon [19,41,42,43,44]. However, the situation is more complex since, as seen in Figure 2b, virtual vibrational spectrometry does not support this unique standardization straightforwardly. As seen in the figure, the Raman spectrum of the basic domain **I** is not of a standard D-G doublet structure but demonstrates a rather well-revealed multiband shape that covers a much wider region than the D-G doublet. The transition to larger domains noticeably narrows the region of the structural spectrum, revealing the growing role of the G band related to *sp*^2^C=C stretching. Conversely, stacking the domain allows for the exhibition of a completely different type of the spectrum structuring, which brings it closer to the standard D-G doublet structure. Thus, the obtained spectra suggest that the shape of the Raman spectrum of a graphene domain depends differently on its size and its stacking. In the first case, an increase in size sharpens the structure of the spectrum in favor of a single G band, while the layering growth leads to a characteristic doublet structure. Since both trends were empirically observed in real “technical graphene” samples (see a profound study of flashed graphenes [44] and references therein), we will return to the feature discussion after analyzing the spectra of necklaced domains from the set shown in Figure 1.

## 4. Vibrational Spectra of Necklaced Graphene Hydrides

Graphene is friendly with hydrogen, which, both in natural and industrial conditions, is one of the first elements seeking to partially inhibit the dandling-bond activity of initially bare graphene domains. A detailed discussion of the graphene hydrogenation, both virtual and experimental, can be found elsewhere [20]. The hydrogen role for natural molecules is largely discussed by the example of natural *sp^2^* amorphous carbons [16–19.45]. Let us consider this point in view of virtual vibrational spectrometry basing on DTs **VI**–**XII** (see Table 1 and Figure 1).

**Figure 3:**
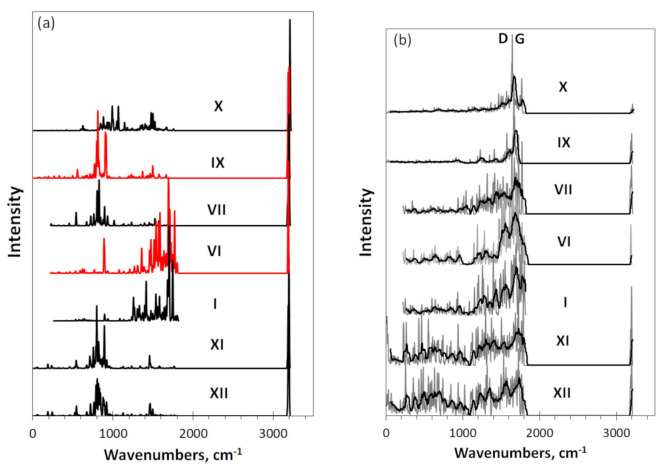
Virtual IR absorption (**a**) and Raman scattering (**b**) spectra of DTs related to necklaced graphene hydride: the reference (5, 5) graphene domain (**I),** the latter with one (**VI**) and 22 (**VII**) hydrogen atoms in the necklace; the (9, 9) and (11, 11) domains, necklaced with 38 and 46 hydrogen atoms, (**IX**) and (**X**), respectively; two-layer (**XI**) and three-layer (**XII**) stacks of **VII**. Digits mark DTs in accordance with Figure 1 and Table 1. UHF AM1 calculations. Two-color plotting is applied to clearly visualize intersected spectral regions.

Virtual IR and Raman spectra of DTs related to necklaced graphene hydrides **VI-XII** are shown in Figure 3. Intensity of each spectrum is normalized per the maximum value, which in Figure 3a is one of bands of the *sp^2^*C-H stretchings. That is why the spectra cannot be compared by intensity that evidently grows when either the hydrogenation proceeds or the graphene domain of the hydride increases. Accordingly, only spectra shapes will be discussed in what follows. Plottings are grouped around the reference spectra of the (5. 5) domain (**I**). Spectra **VI, VII, IX,** and **X**, located above the reference one, reflect changes related to the increase in hydrogen content around the reference domain (**VI** and **VII**) and the size of the latter (**IX** and **X**). Spectra **XI** and **XII** below the references are related to two-layer (**XI**) and three-layer (**XII**) stacks of **VII**.

The IR spectrum **I** of the basic domain was discussed in the previous section. As said, multiband pattern of the spectra is provided with *sp*^2^C=C stretchings, benzenoid ring breathings as well as C-C-C trigonal bendings and puckerings. The absolute value of the spectrum intensity is low due to homopolar valence bonds with nil static moment [40]. The addition of only one hydrogen atom to the domain circumference drastically reduces the carbon atom contribution to the spectrum **VI**, making it small on the background of the intense band of the only one *sp*^2^C-H stretching at 3200 cm^−1^ that clearly dominates. Simultaneously, the origin of a new contribution related to the *sp*^2^C-H bending at ~900 cm^−1^ is clearly seen. When moving from spectrum **VI** to **VII**, related to totally hydrogenated *peri*pentacene C_66_H_22_, the contributions of the carbon part is drastically suppressed, remaining only at a low-intense multi-band structure of ~1700 cm^−1^, while the region of *sp*^2^C-H bending at 900–1200 cm^−1^ is greatly intensified and extended.

**Figure 4:**
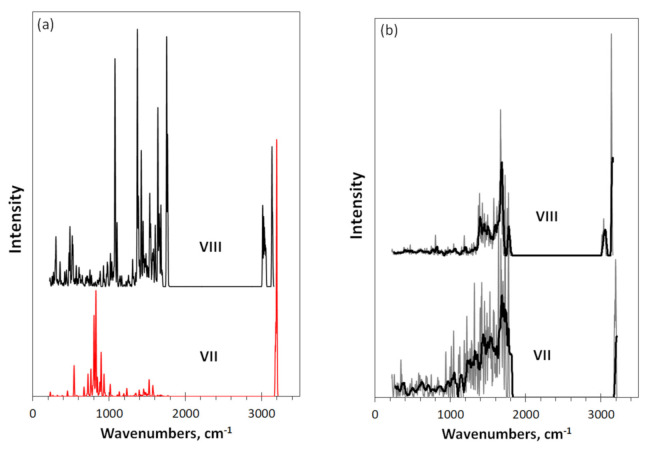
Virtual IR absorption (**a**) and Raman scattering (**b**) spectra of DTs related to necklaced graphene hydrides: (5, 5) domain with 22 hydrogen atoms (**VII**) and 18 methyl units (**VIII**) in the circumference. Digits mark DTs in accordance with Figure 1 and Table 1. UHF AM1 calculations. Two-color plotting is applied to visualize intersected spectral regions.

Comparing spectra **I, VI**, and **VII**, one can see that the fine structure of the carbon atoms spectra is noticeably changed, which was first noted for coronene [2] and is attributed to the effect the added hydrogen atom on the *sp*^2^C=C bonds length and valence angles. To prove the suggestion, Figure 4 presents the IR spectrum of the peripentacene **VII**, hydrogen atoms of which are substituted by methyl units (**VIII**). This action leads to a full reconstruction of the IR spectrum **VII**. Thus, as seen in Figure 4a, in the high frequency region, a single band of individual *sp*^2^C-H stretchings of spectrum **VII** is replaced with two-band spectra of the set of *sp*^3^C-H stretchings of the CH_3_-units. The middle frequency part of the spectrum is filled with C-CH_3_ stretchings as well as C-CH_3_ and CH_3_ internal bendings in the regions of 900–1200 and 1200–1800 cm^−1^, respectively (see Table 2). The latter dominate by intensity thus fully reconstructing the total spectrum shape, which was governed by dominating role of the *sp^2^*C-H stretchings at 3200 cm^−1^.

In Figure 3b, we see that in contrast to the IR absorption, homopolar covalent bonds are favorable for Raman scattering [40]. However, spectra shown in the figure exhibit a rather pronounced participation of hydrogen atoms. In this case, the effect is not connected with hydrogen atoms directly, but results from the influence of the hydrogen presence on the configuration of the *sp*^2^C=C bonds. The latter is quite remarkable and is obviously caused by a particular delocalization of the electron and spin density over the carbon atoms of any honeycomb structure (see [13,26] and references therein). As seen, the presence of hydrogen significantly influences Raman spectrum **I** of the reference (5, 5) domain , starting form one atom in the DT necklace (spectrum **VI**). We observe the appearance of the *sp*^2^C-H stretching band at 3200 cm^−1^ and a considerable reconstruction of the spectrum of *sp*^2^C=C vibrations, particularly, in the 1400–1800 cm^−1^ region. Strong influence of one hydrogen atom on the dynamic of the DT consisting of 66 heavy atoms is highly impressive and was observed for the first time recently [2]. Evidently, the feature is connected with a particular delocalization of the spin electrons density over the graphene domain atoms, making any chemical attachment to the carbon core of the molecules non-local [26].

The necklace of 22 hydrogen atoms corresponds to case (**VII**), when all edge atoms of the graphene domain **I** are once determined (see Figure 1). As seen in Figure 3b, in contrast the IR spectrum **VII**, the Raman spectrum **VII** is not changed drastically, leaving a comparable contribution of the *sp*^2^C-H stretchings with respect to that of molecule **VI** and evidencing the main role of the *sp*^2^C=C vibrations, only accompanied the changing in the relative spectral image. At the same time, the substitution of hydrogen atoms with methyl units (spectrum **VIII** in Figure 4b) leads to a full reconstruction of Raman spectra of the molecule. As seen in the figure, nothing else but a complete suppression of the contribution of *sp*^2^C=C vibrations and, instead, visualization of the scattering on the C-CH_3_ modes, can explain the observed change with respect to that of **VII**. Apparently, the explanation of the feature is the following. Hydrogen atoms of DT **VII** are directly attached to the graphene domain, while those of CH_3_ units are on a distant spacing through C-C bonds in DT **VIII**. The feature may explain why *sp*^2^C-H vibrations are dissolved in the scattering pool of the *sp*^2^C=C modes in the first case, while *sp*^3^C-H ones become dominating scatterers in the second. This is an evident revealing of the difference related to the non-local and local attachments of hydrogens. The feature is one more exhibition of unique physicochemical properties of graphene molecules and deserves a further study.

**Figure 5:**
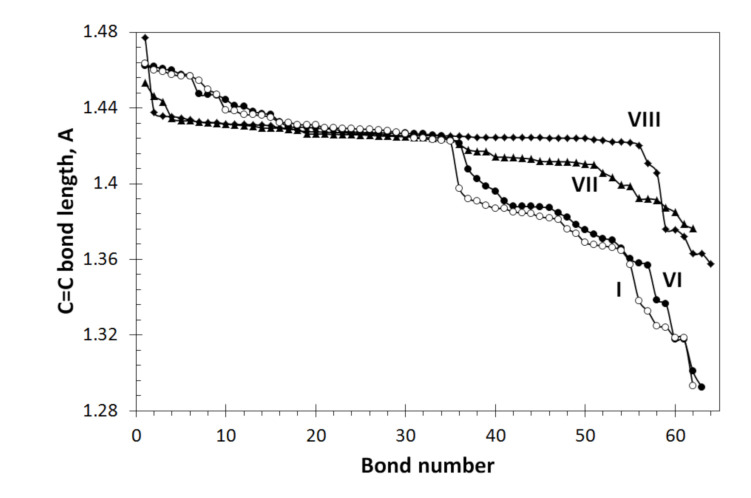
Z→A distribution of the *sp*^2^C=C bond length in necklaced graphene hydrides DTs: (5, 5) graphene domain (**I**); the same domain with one (**VI**) and 22 (**VII**) hydrogen atoms as well as 18 methyl units (**VIII**). Digits mark DTs in accordance with Figure 1 and Table 1. UHF AM1 calculations.

Additionally, changes in the Raman spectra of *sp*^2^ C=C vibrations of DTs **I**, **VI**, **VII**, and **VIII** are evidently connected with the difference of the *sp*^2^C=C bonds pool in the relevant cases. Figure 5 shows the distributions of the DTs *sp*^2^ C=C bonds over their length. As seen in the figure, the addition of the only hydrogen atom to the DT necklace causes quite remarkable change in the C=C bond distribution (plottings **I** and **VI**). Expectedly, 22 hydrogen atoms violate the distribution much stronger (**VII**) and their substitution with methyl units (**VIII**) significantly strengthens the changes. Noteworthy is the noticeable ordering in the bond pool of DTs **VII** and **VIII**, which undoubtedly explains the change in the structure of the Raman spectrum of these objects in comparison with spectrum **I**.

The shape of Raman spectra of graphene hydrides in Figure 3b is of a particular interest, which was partially discussed in the previous Section. Empirically. Raman spectra of nanosized graphene-based specie are characterized with a widely known standard features that is the D-G band doublet. The two components are located in the 1200–1700 cm^−1^ region; the intensity ratio R=IGID and the bands width largely vary and are usually used to judge the degree of ideality of the graphene domains structure [41,42,43,44,45,46,47]. The first attempt to understand the universal standard of this dual-band structure was made in the previous section based on the results of analysis of the Raman spectra of a set of bare graphene domains. It has been found that the size of the domains and the stacking of the latter determine whether the spectrum is single-band or dual-band. The results presented in Figure 3b allow us to make this conclusion more convincing. As can be seen from the figure, the replacement of the (5, 5) domain with either (9, 9) (**IX**) or (11, 11) (**X**) one leads to the emergence of a clearly pronounced single-band spectrum, similar to that of bare domains. The presence of hydrogen atoms does not interfere with this transformation of the spectra. Conversely, stacking of domains, in this case belonging to DT **VII**, makes it possible to trace the formation of the doublet D-G structure using the example of DTs **XI** and **XII**. Therefore, Raman spectra of bare domains and domains with hydrogen necklaces behave similarly, exhibiting both single-band and dual-band structuring when either growing the domain size or stacking the latter. This trend is highly consistent with experimental observation of a large set of flashed graphenes [44].

## 5. Vibrational Spectra of Necklaced Graphene Oxides

A large variability of the spectral signature of the above graphene hydrides, depending on their structure, indicates the practical impossibility of determining the standard form of their vibrational spectra of both IR absorption and Raman scattering. Equally impossible, strictly speaking, is the definition of a standard DT representing this class of species. Nevertheless, certain possibilities of such a standardization, aimed at establishing the “hydrogen sign” in a “technical graphene”, are possible. In this case, we mainly discuss IR absorption spectra, since this effect is less pronounced in the Raman ones. As for the former, according to the data presented in Figure 3a, the characteristic hydrogen spectrum is almost completely formed already on the example of DT **VII**. It is the case when the graphene domain is of the (5, 5) type and the one-atom filling of dangling carbon bonds at the domain boundaries is reached. As can be seen from the figure, the hydrogen spectra of DTs **IX**–**XII** bear clear characteristic features of the DT **VII** spectrum. Furthermore, and retaining the basic (5, 5) domain, while replacing hydrogen atoms with methyl groups, we obtain the characteristic form of a “methyl-units sign” in necklaced graphene hydrides shown in Figure 4a. Extending the established hydrogen tendencies to other heteroatoms, we place the (5, 5) domain in the foundation of DTs related to other polyderivatives, including oxygenated (DT **XIII**), hydroxygenated (DT **XIV**), and carboxylated (DT **XV**) necklaced graphene oxides (Figure 1).

**Figure 6:**
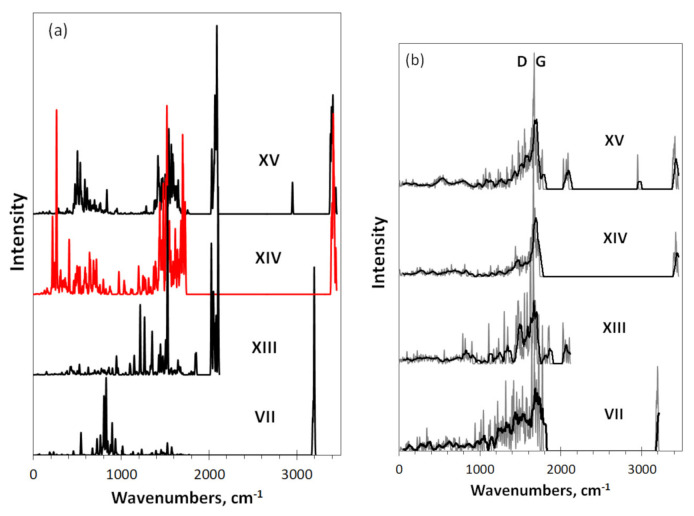
Virtual IR absorption (**a**) and Raman scattering (**b**) spectra of DTs related to necklaced graphene oxides based on (5, 5) graphene domain: the reference graphene hydride (**VII**), graphene oxides with 22 oxygen atoms (**XIII**) as well as 22 hydroxyl (**XIV**) and 18 carboxyl units (**XV**) in the circumference. UHF AM1 calculations. Digits mark DTs in accordance with Figure 1 and Table 1. UHF calculations. Two-color plotting is applied to visualize intersected spectral regions.

Virtual IR absorption (a) and Raman scattering spectra of the corresponding DTs are presented in Figure 6. Not the spectra of the graphene domain itself (**I**), but those of necklaced graphene hydride **VII** are taken as the reference ones to facilitate the job. As seen in Figure 6a, similarly to hydrides, IR spectra of the oxides are related to their necklaces and are well coherent with the corresponding spectral transformation that is characteristic for the spectra of numerous organic molecules when moving from pure oxygen to hydroxylic and carboxylic addends (see [11.12] for details). As seen in Figure 6a, the substitution of hydrogen atoms (**VII**) with oxygens (**XIII**) causes the appearance of a multiband structure in the region of C=O stretchings at ~2000 cm^−1^ and C=O deformations at 1200–1800 cm^−1^. The former dominate in the spectrum thus determining its shape. Passing to hydroxylated graphene (**XIV**) is followed with the disappearance of the C=O stretchings and their substitution with C-O(H) stretchings at 1400–1800 cm^−1^, C-OH bendings at 400–800 cm^−1^, and C-OH torsions at 200 cm^−1^. Simultaneously, a set of CO-H stretchings at 3400 cm^−1^ complete the spectrum of DT **XIV** at the high-frequency edge. In the spectrum **XV** of the carboxylated graphene, we expectedly see C-OH bendings at 400 cm^−1^. C-OH and C=O stretchings at 1400 and 2000 cm^−1^, respectively, and characteristic CO-H stretchings at 2900 and 3400 cm^−1^. As seen in the figure, the spectral features are well consistent with the data for other organic molecules, GFs of which are listed in Table 2. Therefore, IR spectra of graphene oxides are quite characteristic and suggest a clearly seen “oxygen sign” in necklaced graphene molecules.

In contrast to the IR absorption. Raman scattering is not related to the molecules’ necklaces and, as in the case of graphene hydrides, are provided mainly with *sp*^2^C=C stretchings of the graphene domain, which are added with C=O stretchings of **XIII** and **XV** oxides. The difference of the C=C part is evidently caused by the domain structure distortion by the necklace content.

## 6. Discussion of the Results Obtained with Respect to *sp*^2^ Amorphous Carbons

Despite that the virtual spectrometry is not strongly directed to the fitting of the virtual and empirical spectra, there is a strong temptation to check trends observed in the course of the computational experiment. Obviously, “graphene domain”, “hydrogen”. and “oxygen” signs of the necklaced graphene molecules vibrational spectra are the main trends revealed by the virtual spectrometry in the current study. The use of the obtained trends for the analysis and interpretation of experimental spectra faces the difficulty of the absence of a comparison object. The point is that, as noted in the Introduction, the bare and heteroatom-necklaced graphene molecules are not observed as individual objects: the former—due to their high reactivity, and the latter—due to high efficiency of stacking and subsequent agglomeration. In reality, the molecules exist as basic structure units of solids of multilevel structure that present numerous kinds of *sp*^2^ amorphous carbons [15]. Each of these solids has its own rich history of origin, synthesis or production. Accordingly, each solid presentation is followed with a large host of questions concerning the spatial structure homogeneity and necklace chemical composition. Determining these parameters is a complex and time-consuming procedure [16,17,18,19]. At the same time, these materials, such as carbon black, flash graphene, reduced graphene oxide, shungite carbon, and so on, are in great demand from the modern high-tech industry. In these conditions, express analysis methods are extremely demanded, which could make it possible to quickly and reliably characterize the amorphic under request. Until now, the most common optical method has been Raman scattering. However, as shown in this work, Raman spectra are lowly sensitive to the chemical composition of the necklaced graphene molecules and the standard structure of the D-G doublet, which is experimentally observed in the overwhelming majority of cases, is practically not informative. In contrast to the Raman spectrum, the IR spectra are consistently characteristic, which could be the basis of a reliable express method. Let us see how it can work in practice.

To do this, first it is necessary to construct DTs of the amorphic under study. Diverse experimental studies, recently performed for a selected set of *sp*^2^ amorphous carbons (ACs) [16,17,18,19], have allowed for the suggestion of reliable molecular models of their BSUs. The first group of models presents nanosized necklaced graphene molecules constructed on the basis of the (5, 5) graphene domain because of the proportionality of the BSU empirically determined sizes with those of the domain. The structures are accepted to be characteristic for natural *sp^2^* amorphous solids involving anthracite, anthraxolite, and shungite carbon [17]. Virtual IR absorption spectra of the DTs, obtained by use of HF Spectrodyn, were compared with experimental DRIFT data successfully enough [17,48]. The second group of the required DTs has to be constructed on the basis of the (9, 9) graphene domain. Two participants of the group, being the representatives of two kinds of the rGO produced, namely TE-rGO (**XVI**) [17.22] and Ak-rGO (**XVII**) [17.23], are shown in the last row of Figure 1.

**Figure 7:**
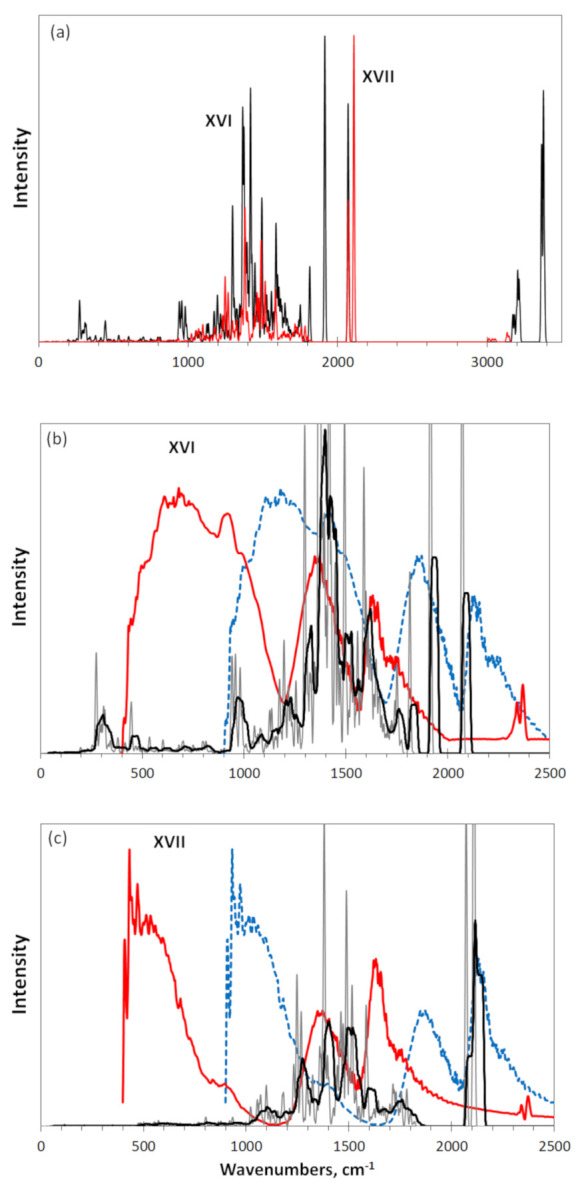
Virtual IR absorption spectra of DTs **XVI** (black) and **XVII** (red) (**a**); a comparison of IR spectrum of DT **XVI** and DRIFT spectrum of TE-rGO (red and blue) (**b**); the same as in (**a**), but for DT **XVII** and Ak-rGO (**c**). Digits mark DTs in accordance with Figure 1 and Table 1. UHF AM1 calculations. Experimental spectra are adapted from [17].

The HF-Spectrodyn virtual IR spectra of these TDs alongside with experimental DRIFT spectra of the bodies are shown in Figure 7. As seen in Figure 7a, the general parts of both virtual spectra, including a rich multiband structure in the region of 1000–1900 cm^−1^ and narrow band at 2100 cm^−1^, is much in common despite a considerable difference in the chemical compositions of the DTs necklaces. Evidently, the first region is well consistent with a composition of CH_3_ units, presenting C-CH_3_ stretchings and bendings as it was discussed relating to the **VIII** spectrum in Figure 4a. However, methyl units are the hydrogen containing elements of DT **XVI** only, while this role in the case of DT **XVII** is given to methylene ones. Apparently, a general coincidence of the GFs for both units (see Table 2) as well as a large number of the relevant vibrational modes provide strong similarity of this part of the obtained virtual IR spectra, making them practically indistinguishable for the two units. As for the small band group around 2100 cm^−1^, the previous study of the virtual spectra of graphene oxides allows for the reliable attribution of it to the presence of quinone units in the circumference of both DTs. Besides the two groups, two weak bands at ~3100 cm^−1^ of the DT **XVII** spectrum should be attributed to *sp*^3^C-H stretchings, while spectrum **XVI** involves an intense band at 1700–1900 cm^−1^ related to C=O stretchings of the peripheral ether and lactone groups as well as *sp^2^*C-H and CO-H stretchings at 3200 cm^−1^ and 3400 cm^−1^, respectively. A more detailed description of characteristic GFs related to *sp*^2^ amorphous carbons is given elsewhere [17].

A comparison of the virtual spectrum **XVI** and experimental DEIFT spectrum of TE-rGO is shown in Figure 7b. As seen in the figure, at first glance, the real object spectrum (red potting) differs drastically from the virtual one. However, once upshifted on 500 cm^−1^, three-band spectrum (blue plotting) well correlates with similar three-band virtual one, reliably supporting atomic configuration of the DT **XVI**. Naturally, it is impossible to speak about a complete adequacy of the proposed model to the real BSU structure, but the fact of reproduction of the main components of the latter is obvious. In the current case, the evidence, provided with the INS [16] as well as DRIFT and XPS [17] spectra, is supported with virtual spectrometry.

A completely different situation is presented in Figure 7c. In contrast to the previous case, the experimental spectrum of Ak-rGO, which differs significantly from that for TE-rGO, is also different from the virtual spectrum of DT **XVII**. As can be seen, a similar upshift does not reveal any similarity between the real and virtual spectra, except for the band at 2100 cm^−1^. This band is perhaps the only evidence of the presence of quinones in the structure of both real Ak-rGO BSU and DT **XVII**. As for the hydrogen-containing part, none of the forms of the considered C-H vibrations can be associated with the experimental spectrum in the region of 400–1000 cm^– 1^. Downshifted, this part of the spectrum is formed by other heteroatoms, mainly oxygen, the structural composition of which remains to be determined. Perhaps, hydroxylic units in this case will play the most important role. As follows from Figure 4a, the participation of hydroxyls in the formation of the necklace of graphene molecule leads to the appearance of an intense downshifted spectrum in the region of 600–1200 cm^−1^. In contrast to the previous case, virtual spectrometry does not confirm the conclusions drawn on the basis of the analysis of INS, DRIFT, and XPS spectra of Ak-rGO [17].

**Figure 8:**
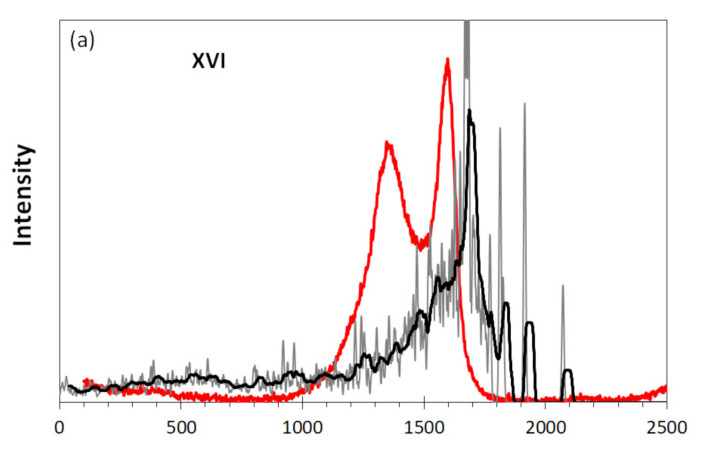

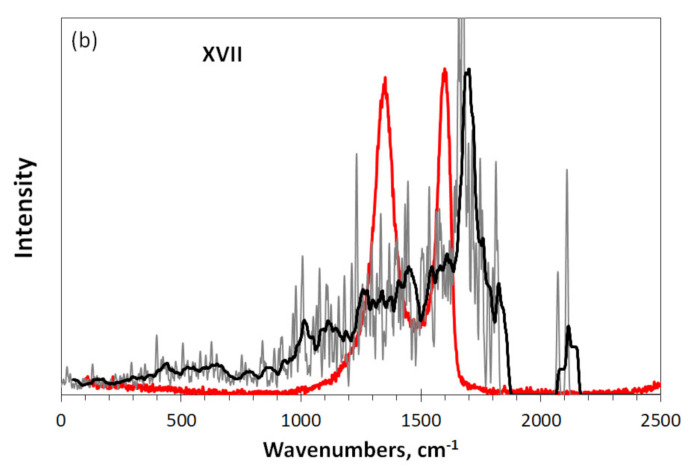
Comparison of virtual and experimental Raman spectra: DT **XVI** and TE-rGO (**a**) and DT **XVII** and Ak-rGO (**b**). Digits mark DTs in accordance with Figure 1 and Table 1. UHF AM1 calculations. Experimental spectra are adapted from [18].

A comparison of experimental and virtual Raman spectra related to TE-rGO and Ak-rGO is presented in Figure 8. In contrast to IR spectra, both experimental and virtual spectra of the two species are quite similar, exhibiting a characteristic D-G doublet structure empirically (red plotting), with an obvious domination of a single G band in the latter (black plotting) virtually. The discussion of the spectra details can be found elsewhere [18]. The two virtual spectra are not only similar for the considered case, but clearly demonstrate the same trend with respect to virtual spectra **IV** and **V** in Figure 2b as well as to spectra **IX** and **X** in Figure 3b. The feature allows for the conclusion that one-band transformation of Raman spectra is typical when the graphene domain size of individual molecules is approaching 15 nm [18]. Therewith, the corresponding spectra depend weakly on the chemical composition of the corresponding necklaces and evidence only the origin of coherent phonon state in the domain generated with coherent *sp*^2^C=C stretchings.

## 7. Conclusions

Virtual vibrational spectrometry is at its beginning. Nevertheless, the findings provide compelling evidence of a new dimension of research. Perhaps the age-old dream of all spectroscopists to establish a direct structure–spectral image relationship is coming true. With each new object, this becomes more and more obvious. This spectrometric technique is free of experimental uncertainty related to sample preparation and the competence of the experimental technique in use and provides the innocent consideration of the objects. Moreover, it allows for the manipulation of the latter in any wished way, restricted only with the facility of the computational programs in use. Digital twins instead of real objects and computational software instead of real spectrometers are real instruments of the new spectrometry that provide the realization of the dream. Comparative analysis leading to the revealing of trends, common to both virtual and experimental spectra, is the strong point of the spectrometry. The unavoidable discrepancy of obtained individual virtual spectra and experimental data is its vulnerability. Therefore, trends, but not individual spectral signatures, are the spectrometry’s aim.

Based on HF Spectrodyn, the approach has positively shown itself on the example of *sp*^2^ carbon clusters formed by polycyclic benzenoid-fused hydrocarbons [2], fullerene C_60_ and its isomers [4], and fullerene C_60_ dimers [5]. Reliable attribution of vibrational spectra of the molecules to standard covalent bonds subjected to particular structural and atomic-content affects, removal of the problem of the C_60_ “silent” vibrational modes, exhibition of reasons of the only empirical structure from numerous C_60_ isomers, elicitation of the C_60_ dimers topochemical nature, explanation of high sensitivity of the dimers empirical structure to the preparation conditions—this is a short list of the tasks that have been thus far successfully solved. A high efficiency of the spectrometer allows for dealing with large molecules, both closed- and open-shell ones, which makes it possible to turn to such complex objects as necklaced graphene molecules. This name refers not to molecular individuals, but to a vast class of substances whose individual images do not have a standard composition and structure, the determination of which in the framework of the usual inverse spectral problem is not effective. Obviously, it is these DT objects, to which the virtual spectrometry is the most favorable and effective. The first results obtained in this area are presented in this article.

Evidently, the class of necklaced graphene molecules is practically endless starting from bare *sp^2^* honeycomb sheets of different size and shape (graphene domains) and ending with countless number of the domain polyderivatives involving hydrides, oxides, oxyhydrides, nitrides, and many others. The polyderivatization concerns only the domain circumference, thus giving the view of the bodies as a graphene domain with a heteroatom necklace. All the objects under study were presented as the relevant DTs of necklaced graphene molecules.

The following trends have been revealed in due course of the experiment.

Supporting the previous results [2.4.5], vibrational spectra of the bodies are determined by the pool of covalent bonds, among which *sp*^2^C=C bonds are homopolar while all the others are heteropolar.

IR and Raman spectra of the bodies are provided with different components of the bonds pool. Covalent homopolar *sp*^2^C=C bonds do not possess a static dipole moment due to their IR activity arising from a dynamic mechanism in which the charge is transferred from extended to compressed bonds [40]. Accordingly, IR absorption of graphene domains is extremely weak, while the necklaced graphene bodies show considerable absorption provided with heteropolar bonds and are individually dependent on the chemical composition of the necklaces. In contrast, activity of the Raman scattering is provided with *sp*^2^C=C bonds mainly due to which Raman signatures of bare graphene domains and the relevant necklaced bodies are similar.

IR spectra depend on the size of the body graphene domain rather weakly. In contrast, Raman spectra show a drastic dependence on the linear size and packing of both bare and necklaced graphene domains. When the domain dimension approaches ~15 nm. which is the free path of the high frequency optical phonons in graphene crystal [18], the Raman spectrum of all DTs takes the single-band form similar to quasi-particle one-phonon one of graphene crystal [48].

The necklaced graphene molecules are prone to stacking and agglomeration. The former examined in the current study showed a practical tolerance of IR spectra, but a drastic change of the Raman ones. The latter, once multiband for individual bodies, take on a dual-band form similar to the standard empirical D-G doublets when stacking.

This finding removes the veil from the mystery of the universal standard of Raman spectra of graphene-containing materials of various origins. A huge number of spears were broken when trying to explain this standard form of Raman spectrum, regardless of the wide variability of the structure and chemical composition of the studied materials. The above analysis of the Raman spectra of necklaced graphene molecules opens up a new possibility of linking this fact with the stacked nature of the investigated solids. The transformation of a multiband spectrum of an individual molecule into a D-G doublet can be qualitatively described by the amplification of the D band with respect to the G one. Obviously, the interaction between molecules in adjacent layers is the reason. This interaction expectedly affects the dynamics of the *sp*^2^C=C bonds in layers that are extremely flexible. However, more important seems to be another thing—a strong dynamical coupling between the layers. Actually, the interlayer distance in stacks of graphene materials is of ~3.4 Å, once equalizing the van der Waals diameter of carbon atoms. Evidently, the out-of-plane vibrations, which are typical for multilayered graphene [49,50,51,52], can markedly shorten the interlayer C^….^C distance approaching it to the maximum length of the *sp*^3^ C bond of ~2.5 Å [53]. Obviously, stable single *sp*^3^C-C bonds between the layers are not formed, while a dynamic imitation of long C-C bonds between the layers may occur. The frequencies of corresponding *sp*^3^C-C stretching expectedly fill the interval that coincides with the D band region. Since the intensity of the out-of-plane phonon D bands in multilayered graphenes significantly grows when the nanostructuring of the latter effectively proceeds [51,52], the linear dimensions of the studied DTs below 15 nm strongly favor the amplification of the D band intensity in stacks of necklaced graphene molecules. Therefore, the presence of a D-G doublet in Raman spectrum of any nanostructured graphene material evidences that the size of the corresponding BSUs is less than 15 nm, while the units themselves are packed in stacks. A particular pattern of the D-G dual band is a rich repository about the package character, intuitively mentioned and discussed previously [18]. Undoubtedly, this conclusion awaits further confirmation.

## Figures and Tables

**Table 1:** Identifying criterion parameters of the odd electron correlation in graphene molecules ^1^.

DT Number	Chemical Formula	Point Symmetry	Number of Atoms	$\mathrm{Spin}\;\mathrm{Multiplicity}\;\Delta N_{\alpha \beta }$ **^2^**	$N_{D}$, *e*	$\Delta {\hat{S}}_{U}^{2}$
Bare graphene domains						
**I**	C_66_	$D_{2h}$	66	triplet	34.2	18.1
**II**	C_132_	$C_{s}$	132	triplet	67.6	34.8
**III**	C_198_	$C_{1}$	198	triplet	101.5	51.8
**IV**	C_190_	$C_{2}$	190	singlet	76.2	38.1
**V**	C_276_	$C_{1}$	276	singlet	95	47.5
Necklaced graphene hydrides						
**VI**	C_66_H	$C_{2v}$	67	doublet	33	16.8
**VII**	C_66_H_22_	$D_{2h}$	88	triplet	14.2	8.1
**VIII**	C_66_ (CH_3_)_18_	$D_{2h}$	138	singlet	20	10
**IX**	C_190_H_38_	$C_{2}$	228	singlet	44.6	22.3
**X**	C_276_H_46_	$C_{1}$	322	triplet	56.1	29.1
**XI**	C_132_H_44_	$D_{2h}$	176	triplet	29	15.5
**XII**	C_198_H_66_	$C_{2h}$	264	triplet	40.4	21.2
Necklaced graphene oxides						
**XIII**	C_66_O_22_	$C_{1}$	88	singlet	16	8
**XIV**	C_66_ (OH)_22_	$C_{1}$	110	singlet	17.8	8.9
**XV**	C_66_ (COOH)_22_	$C_{1}$	146	singlet	20	10
Necklaced graphene oxyhydrides						
**XVI**	C_198_H_66_O_20_	$C_{1}$	236	triplet	55.1	27.9
**XVII**	C_190_H_27_O_11_	$C_{1}$	228	doublet	58.4	29.6

^1^ Radicalization of the studied molecules is characterized by the number of effectively unpaired electrons $N_{D}\;$ and spin contamination $\Delta {\hat{S}}_{U}^{2}$ [13]. ^2^ Formal spin multiplicity is determined by the difference $\Delta N_{\alpha \beta }=N_{\alpha }-N_{\beta }$. $N_{\alpha }$ and $N_{\beta }$ are the numbers of electrons with spins α and β, respectively.

**Table 2:** Standard group frequencies of aromatic molecules required for the fractional analysis of vibrational spectra of amorphous carbons.

Wavenumbers, cm^−1^		Group Frequencies ^1^					
Exper	UHF Expect	*sp*^2^(C, C) ^2^	*sp*^2^(C-H) ^2^	*sp*^2^(C-H) and (C, CH_2_) ^3^	*sp*^3^(C-H) and (C, CH_3_) ^4^	(C, O) and (C, OH) ^5^	(C, O-C)
400–700	700–1000	404 δ *op* C-C-C 606 δ *ip* C-C-C	-	711 ρ CH_2_	210 *r* CH_3_ 344 δ CH_3_	458 τ COH	605 δ C-O
700–1200	1200–1700	707 δ C-C-C puckering 993 ring breathing 1010 δ C-C-C trigonal	673 δ *op* in phase 846 δ *op* C_6_ libration 967 δ *op* 990 δ *op* trigonal 1037 δ *ip* 1146 δ *ip* trigonal 1178 δ *ip*	948 ρ CH_2_	900 ν C-CH_3_ 1041 ρ CH_3_	960 ν C-O(H) 1158 δ C-O(H) 1284 ν C-O(H)	970 ν C-O 1260 ν C-O
1200–1600	1700–2100	1309 ν C=C Kekule 1482 ν C=C 1599 ν C=C	1350 δ *ip* in phase	1409 δ internal CH_2_	1333 δ CH_3_ 1486 δ internal CH_3_	1511 ν C=O	-
1600–1900	2100–2400	-	-			1500–1700 ^6^ ν C=O	
2800–3200	3200–3600	-	3056 ν C-H 3057 ν trigonal C-H 3064 ν C-H 3073 ν in phase C-H	3114 ν CH_2_	2950 ν CH_3_	3400 ν OH	-

^1^ Greek symbols ν, δ, ρ, *r*, τ mark stretching, bending, rocking, rotational, and torsion modes, respectively. ^2^ GFs notifications of fundamental vibrations of benzene molecule [30]. ^3^ GFs notifications of fundamental vibrations of benzyl radical [31,32]. Hereinafter, GFs, additional to the benzene pool of vibrations, will be shown only. ^4^ GFs notifications of fundamental vibrations of toluene [33,34]. ^5^ GFs notifications of fundamental vibrations of *p*-benzosemiquinone [35]. ^6^ GFs notifications of fundamental vibrations of a large collection of organic molecules [11,12].
